# Evaluation of an ImmunoPET Tracer for IL-12 in a Preclinical Model of Inflammatory Immune Responses

**DOI:** 10.3389/fimmu.2022.870110

**Published:** 2022-05-11

**Authors:** Nerissa T. Viola, James E. Glassbrook, Jhansi R. Kalluri, Justin B. Hackett, Madison N. Wicker, Joshua Sternberg, Heather M. Gibson

**Affiliations:** ^1^Department of Oncology, Karmanos Cancer Institute, Wayne State University, Detroit, MI, United States; ^2^Department of Biochemistry Microbiology and Immunology, Wayne State University, Detroit, MI, United States; ^3^Cancer Biology Graduate Program, School of Medicine, Wayne State University, Detroit, MI, United States

**Keywords:** Interleukin-12, PET imaging, immunotherapy, inflammation, radioactive tracer

## Abstract

The immune cytokine interleukin-12 (IL-12) is involved in cancer initiation and progression, autoimmunity, as well as graft versus host disease. The ability to monitor IL-12 *via* imaging may provide insight into various immune processes, including levels of antitumor immunity, inflammation, and infection due to its functions in immune signaling. Here, we report the development and preclinical evaluation of an antibody-based IL-12-specific positron emission tomography (PET) tracer. To mimic localized infection and stimulate IL-12 production, BALB/c mice were administered lipopolysaccharide (LPS) intramuscularly. [^89^Zr]Zr-DFO-αIL12 tracer was given one hour post LPS administration and PET images were taken after 5, 24, 48, and 72 hours. We observed significantly higher uptake in LPS-treated mice as compared to controls. Biodistribution of the tracer was evaluated in a separate cohort of mice, where tracer uptake was elevated in muscle, spleen, lymph nodes, and intestines after LPS administration. To evaluate the utility of [^89^Zr]Zr-DFO-αIL12 as an indicator of antigen presenting cell activation after cancer immunotherapy, we compared PET imaging with and without intratumoral delivery of oncolytic adenovirus expressing granulocyte-macrophage colony-stimulating factor (Adv/GM-CSF), which we have shown promotes anti-tumor immunity. BALB/c mice were inoculated orthotopically with the mouse mammary carcinoma line TUBO. Once TUBO tumors reached a volume of ~50 mm^3^, mice were treated with either three intratumoral injections of 10^8^ PFU Adv/GM-CSF or vehicle control, given every other day. Upon the last dose, [^89^Zr]Zr-DFO-αIL12 was injected intravenously and 72 hours later all mice were imaged *via* PET. Tumor-specific uptake of [^89^Zr]Zr-DFO-αIL12 was higher in Adv/GM-CSF treated mice versus controls. Tissues were harvested after imaging, and elevated levels of macrophages and CD8^+^ T_c_ cells were detected in Adv/GM-CSF treated tumors by immunohistochemistry. We validated that IL-12 expression was induced after Adv/GM-CSF by qRT-PCR. Importantly, expression of genes activated by IL-12 (IFNγ, TNFα, and IL-18) were unaffected after IL-12 imaging relative to mice receiving an IgG control tracer, suggesting the tracer antibody does not significantly disrupt signaling. Our results indicate that targeting soluble cytokines such as IL-12 by PET imaging with antibody tracers may serve as a noninvasive method to evaluate the function of the immune milieu *in situ*.

## Background

The dynamic nature of the immune system presents interesting challenges for the monitoring of various disease states. Imaging modalities have been employed to address this problem by providing non-invasive, real-time visualization of the immune compartment. Positron emission tomography (PET) is one such approach in which a radioisotope is bound or conjugated to a tracer molecule, permitting evaluation of a specific target or cellular pathway. Clinically, the most common PET radiotracer is F-18 fluoredeoxyglucose (FDG), a glucose analog containing ^18^F which is used to identify tissues with high metabolic activity, such as cancer. FDG PET has been proposed as a method to monitor immune activity after cancer immunotherapy, however the lack of specificity to the immune compartment poses a challenge (1–4). To more specifically evaluate immune function, PET imaging technologies are currently under development for a variety of soluble immune signaling targets, including granzyme B, TGFβ, IL-1β, IL-2, TNFα, and IFNγ (5–9). Many of these approaches utilize monoclonal antibodies (mAb) to deliver the radionuclide to its target. Interleukin-12 (IL-12) plays important roles in inflammation, infection, and anti-tumor immunity, making for an attractive imaging target. Detection of IL-12 *via* PET may serve as a useful diagnostic method for monitoring response to immunotherapy, infection, as well as both acute and chronic inflammatory conditions.

The IL-12 signaling family consists of IL-12, IL-23, IL-27, IL-35, and IL-39 (10). Broadly speaking, this family is related based on shared subunits and/or receptors, however each of these cytokines play distinct and nuanced roles in immune function. IL-12 is an essential cytokine that can modulate both the innate and adaptive immune response (11). It has been widely reported to be involved in immune signaling associated with cancer, autoimmunity, and graft versus host disease (10, 12). IL-12 is a pro-inflammatory heterodimeric cytokine (subunits p40, p35) primarily produced by antigen presenting cells (APCs) such as dendritic cells, macrophage, and other monocytes. The p35 subunit is thought to be regulated at the protein translation level based on the amount of expressed p40 subunit, but expression of the p40 subunit is governed by several transcription factors including NF-κB family members, CCAAT enhancer-binding protein β, Ets2, PU.1 (Spi1, Sfpi1), and interferon regulatory elements (IRF family) (13, 14). These subunits are shared by the family members IL-23 (p40) and IL-35 (p35). This is an important consideration when attempting to detect and/or image multimeric proteins, such as IL-12. To ensure specificity to the IL-12 heterodimer, the tracer must target the complex as a whole (p70).

IL-12 binds cell surface heterodimer receptors IL-12Rβ1/2, bringing together intracellular Tyk2 and Jak2, facilitating the phosphorylation of Stat4. Phosphorylated Stat4 dimerizes and acts as a transcription factor, allowing for the upregulation of several key immunoregulatory genes. IL-12 was first discovered in 1989, and originally described as “natural killer cell stimulatory factor” (NKSF). While this moniker was eventually dropped, the group described the addition of purified IL-12 to peripheral blood lymphocytes inducing IFNγ production and increasing cytotoxicity of NK cells (15). CD4^+^ T cells, when exposed to IL-12 in culture prior to TCR stimulation are generally driven toward a T_h1_ phenotype, though their cytokine expression profiles include both T_h1_ and T_h2_ associated cytokines, including IFNγ, TNFα, IL-13, IL-4, and IL-10 (16). Enhanced cytotoxicity and upregulation of IFNγ and TNFα is also observed in CD8^+^ T_c_ exposed to IL-12 prior to TCR signaling (17, 18). In addition, this treatment selectively activated MAPKs and AKT signaling in CD8^+^ T_c_ without activating TCR signaling molecules more proximal to the TCR signaling event (18). IL-12 production is crucial for the recruitment and effector functions of cytotoxic NK cells, T_h1_ cell differentiation, and CD8^+^ T_c_ function.

IL-12 also plays various roles in the context of bacterial, viral, parasitic, and fungal infections. Induction of IL-12 production can be triggered *via* the recognition of pathogen associated molecular patterns (PAMPs) through pattern recognition receptors (PRR). These include but are not limited to toll like receptor (TLR) signaling such as TLR4/9, recognizing lipopolysaccharide (LPS) or unmethylated CpG dinucleotides (CpG-DNA) respectively (19).

In the context of the tumor microenvironment (TME), IL-12 has been shown to inhibit immunosuppression by the myeloid compartment. Tumor associated macrophage (TAMs) are classically known to promote tumor growth and metastasis, but following treatment with IL-12, TAMs exhibit reduced production of tumor promoting factors (IL-10, MCP-1, MIF, and TGFβ) (20). IL-12 treatment was also shown to decrease myeloid derived suppressor cells (MDSC) numbers in the tumor microenvironment, increase survival, and reduce metastasis (21). This is thought to be driven by the sensitization of suppressive cell subsets (such as MDSCs and regulatory T cells) by IL-12, creating an inflammatory TME and promoting CD8^+^ T_c_ killing (22). Thus, detection of an inflammatory TME may prove to be a useful tool for informing clinical decisions related to immunotherapeutic intervention and the prospective localized administration of IL-12.

This body of evidence supports the development of a PET tracer able to noninvasively image IL-12 levels throughout the body, whether to monitor response to immunotherapeutics, to identify sources of infection that elude traditional diagnostic techniques, or to monitor/diagnose patients with chronic inflammatory conditions. Here, we report the development and preclinical evaluation of an IL-12p70-specific immunoPET tracer. We demonstrate that the tracer [^89^Zr]Zr-DFO-αIL12 is capable of detecting IL-12 in various immune-mediated conditions, characterize the biodistribution of the tracer, and show that tracer administration does not impede IL-12 signaling.

## Materials and Methods

### Mice

Age- and sex- matched BALB/c mice (mice roughly 6-8 weeks old, in equal ratios of male:female subjects) were purchased from Charles River Laboratories (Wilmington, MA). All animal procedures were approved by and performed in accordance with the regulation of Wayne State University Animal Care and Use Committee.

### [^89^Zr]Zr-DFO-αIL12 Development

All antibodies (Bioxcell) were buffer exchanged in saline prior to conjugation to p-SCN-Bn-deferoxamine (DFO). Antibody tracers were developed following established protocols (23, 24). Both the murine antibody R2.9A5 (αIL-12) and a control non-specific rat IgG isotype were conjugated with a 1:5 mole ratio of mAb : DFO in saline at pH ~9 for 1 h at 37°C. Unbound DFO was removed *via* spin column centrifugation (MWCO: 30 kDa, GE Vivaspin 500). ^89^Zr in oxalic acid (3D Imaging, Little Rock, AR) was incubated with the mAb-DFO conjugates at pH ~ 7.2-7.4 at room temperature. Unbound ^89^Zr was removed *via* centrifugation with molecular weight column filters (MWCO: 30 kDa, GE Vivaspin 500) using saline as eluent buffer. [^89^Zr]Zr-DFO-αIL12 was labeled at a specific activity of 259 ± 2.6 MBq/mg (7.01 ± 0.07 mCi/mg) and [^89^Zr]Zr-DFO-IgG was labelled at a specific activity of 258 ± 1 MBq/mg (6.98 ±0.03 mCi/mg). Radiochemical yields of both constructs were >95% as determined *via* radio-instant thin layer chromatography (iTLC, Eckert & Ziegler). Stability of the intact [^89^Zr]Zr-DFO-αIL12 was monitored between 24-96 h post-synthesis in saline at 37°C.

### [^89^Zr]Zr-DFO-αIL12 PET Imaging and Distribution in LPS-Treated Mice

BALB/c mice were given 40 µg LPS in 50 μL 1xPBS intramuscularly (right gastrocnemius) or vehicle control. [^89^Zr]Zr-DFO-αIL12 was administered at 1 h post-LPS treatment with 7.4-9.3 MBq (200-250 µCi) for PET imaging and 0.74-0.925 MBq (20-25 µCi) for tissue distribution. PET imaging (Siemens Concord R4) was acquired at 5, 24, 48 and 72 h. Volumes of interest (VOIs), expressed as %injected dose per gram (%ID/g) of tissue based on instrument calibration, were obtained from the muscle injected with LPS, contralateral muscle (C.L.), spleen, heart, and liver. For tissue distribution studies, tissues collected include both regional and contralateral inguinal draining lymph nodes at 24 h post injection (p.i.) of the radiotracer.

### IL-12 PET of Adv/GM-CSF Treated Tumors

Age-matched BALB/c mice were subcutaneously implanted with 2x10^5^ TUBO cells on the left flank. Tumors are permitted to grow to ~50 mm^3^ (10 days) before treatment with intratumoral injections of oncolytic adenovirus expressing granulocyte-macrophage colony-stimulating factor (Adv/GM-CSF). Adv/GM-CSF was obtained through Svend Freytag (Henry Ford Health System, Detroit, MI) (25). Briefly, 10^8^ plaque forming units (PFU) in 10 μL sterile saline or vehicle control were given every other day for 3 total injections. On the final day of treatment,​intravenous (i.v.) lateral tail vein injections of 6.3-8.5 MBq (170-230 µCi) of either [^89^Zr]Zr-DFO-αIL12 or [^89^Zr]Zr-DFO-αIgG were administered in separate cohorts of treated (Tx) and untreated (UTx) mice. PET images were acquired on Bruker Albira Si PET/CT on PET mode alone at 72 h p.i. VOIs were expressed as %injected dose per volume (%ID/mL) based on instrument calibration.

### Immunohistochemistry (IHC)/Image Processing

Immunohistochemistry (IHC) was performed using a CD86 (1:500, tris, PA5-114995, ThermoFisher), Arg1 (1:1000, citrate, 16001-1-AP, Proteintech), and CD8 antibodies (1:400, tris, D4W2Z, Cell Signaling Tech). Tumors were harvested, formalin fixed, embedded in paraffin, and sectioned at 5 µm. After deparaffinization in xylene and graded EtOH, antigen retrieval was performed using Tris buffer at pH 9 (CD86, CD8) or Citrate at pH 4 (Arg1). Primary antibody incubation was performed overnight at 4°C in a humidified chamber. Secondary antibody incubations and detection *via* alkaline phosphatase per manufacturer instruction (Vector Red AP Kit, SK-5100). Cell enumeration was conducted using Trainable Weka Segmentation, available through the Fiji image processing distribution of ImageJ (26, 27).

### qRT-PCR

Tumor tissues were collected 1 week post Adv/GM-CSF administration and 3 days post [^89^Zr]Zr-DFO-αIL12/[^89^Zr]Zr-DFO-IgG control administration. Tumors were immediately flash frozen in liquid nitrogen and allowed to decay for >10 half-lives in –80°C. RNA was extracted *via* Trizol (Thermo Fisher, Waltham, MA) post homogenization by tissue tearer. cDNA was prepared using ProtoScript II reverse transcriptase (New England Biolabs, MA). Real-time qPCR (RT-qPCR) was performed using iTaq Universal Probes Supermix (Bio-Rad Laboratories, Hercules, CA) using 5 ng cDNA/well and 500 nM primers. Primers include Gapdh (Mm99999915_g1), Arg1 (Mm00475988_m1), IL-12β (Mm01288989_m1), IFNγ(Mm01168134_m1), IL-18 (Mm00434225_m1), TNFα (Mm00443258_m1), and Nos2 (Mm00440502_m1) (Life Tech, Carlsbad, CA). mRNA quantification was reported as difference in cycle threshold (ΔCT) relative to GAPDH.

### Data and Statistical Analysis

Statistical analyses were conducted using Graphpad Prism v. 9.3. Imaging and distribution data (IHC counts, qPCR) were analyzed using Mann-Whitney two-tailed non-parametric t-test with multiple comparisons. Data are presented as the mean ± S.D. unless otherwise noted. A p-value < 0.05 is considered statistically significant.

## Results

### [^89^Zr]Zr-DFO-αIL12 Identifies Inflammation *In Vivo*

**Figure 1:**
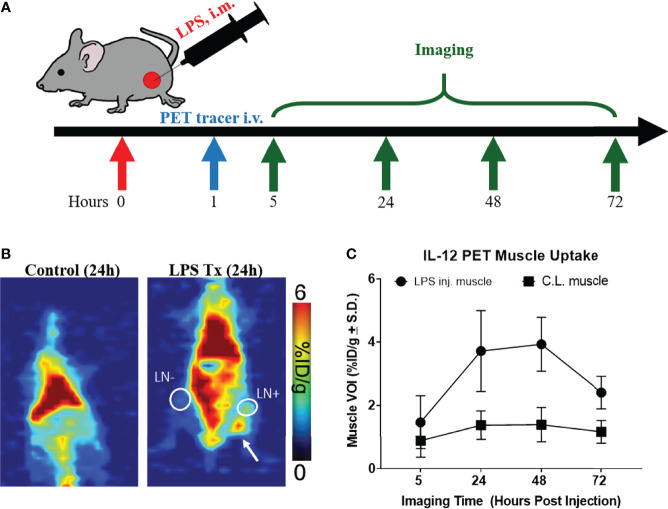
[^89^Zr]-α-IL12 successfully identifies mock infection. **(A)** Experimental design for LPS treatment/imaging. **(B)** Representative planar images of Control/LPS injected mice. LPS → injected tissue. White circle LN+ draining lymph node. White circle LN- contralateral lymph node. **(C)** [^89^Zr]-αIL12 uptake over a 3-day time course.

The rat IgG2b κ mAb R2.9A5 to murine IL-12p70 was labeled with ^89^Zr, using desferrioxamine (DFO) as a chelator, producing [^89^Zr]Zr-DFO-αIL12. Yield and purity of the compound was within the expected range based on previous studies (**Figure S1A**) (24). The radiotracer appears >98% intact after 96 h of incubation at 37°C (**Table S1**). LPS (40 µg LPS in 50 µL PBS) was administered intramuscularly on the right hind leg to mimic infection and stimulate IL-12 production. The dose of LPS, while far below the lethal dose (LD50 = 10 mg/kg body weight (28)), is within the reported range to induce systemic cytokine production (29). One hour post LPS administration, mice received [^89^Zr]Zr-DFO-αIL12 tracer by intravenous injection and PET images were taken 5, 24, 48, and 72 h later to determine the time point with optimal tracer uptake at the LPS injection site and secondary lymphoid organs relative to background (**Figures 1A–C**). Volumes of interest (VOI) drawn on the injection site showed higher tracer accumulation on the LPS-injected muscle at 24 h (3.7 ± 1.3%ID/g, n=5) and 48 h p.i. (3.9 ± 0.8%ID/g) compared to the C.L. muscle (24 h: 1.4 ± 0.4%ID/g, p=0.0047; 48 h: 1.4 ± 0.5%ID/g, p=0.0005) (**Figures 1B, C**). Time-activity curves exhibited decreasing non-specific binding of the radiotracer in the spleen, heart and liver over time (**Figure S1B**).

**Figure 2:**
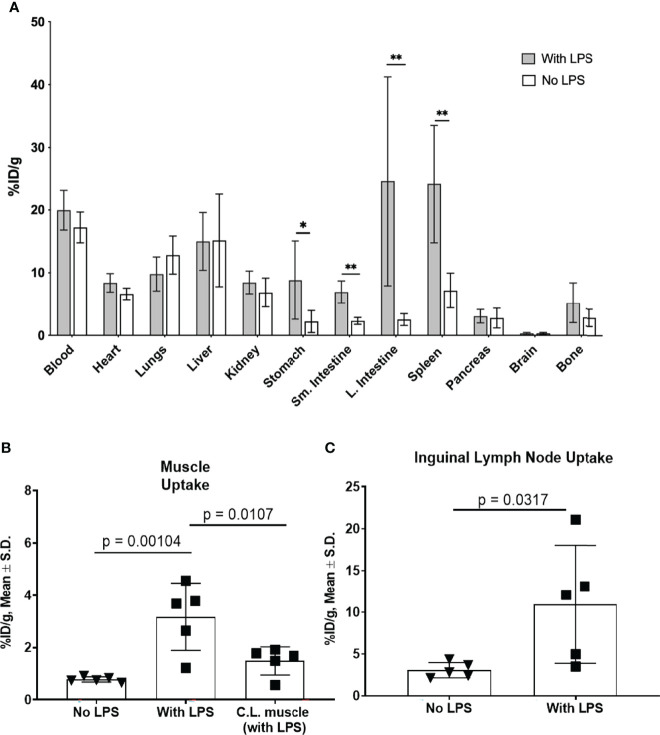
Tracer biodistribution 24 h p.i. with and without LPS administration **(A).** [^89^Zr]-αIL-12 uptake (%ID/g) in select tissues shows significant differences in gastrointestinal tissues and spleen. Tracer uptake in **(B).** lateral (injection site) and contralateral (C.L.) muscle and **(C).** inguinal draining lymph node in LPS-treated versus untreated mice. *p value < 0.05, **p value < 0.01.

In a separate cohort of mice, biodistribution of [^89^Zr]Zr-DFO-αIL12 tracer was performed at 24 h p.i. (**Figure 2A**). Tracer uptake in the LPS-injected muscle (3.2 ± 1.3% injected dose/gram (ID/g)) was significantly higher than muscle tissue from naïve, non-LPS injected control mice (0.8 ± 0.1%ID/g, p=0.0079) (**Figure 2B**). The C.L. muscle from LPS-treated mice also exhibited tracer uptake (1.5 ± 0.5%ID/g) which while lower, was not statistically significantly different than paired ipsilateral muscle. This may be due to slightly elevated circulating IL-12 levels in the bloodstream after LPS administration. Both draining (DLN: 11.0 ± 7.1%ID/g, p=0.038) and C.L. lymph nodes (CLN: 9.7 ± 4.4%ID/g, p=0.0085) displayed higher accumulation of the radiotracer versus lymph nodes of the untreated mice (2.8 ± 1.0%ID/g), reflecting the systemic response to LPS (**Figure 2C**). Furthermore, elevated splenic tracer accumulation was observed in LPS-treated compared to naïve mice (24.1 ± 7.2%ID/g versus 7.2 ± 2.8%ID/g, p=0.0047). The probe also exhibited high accretion in the spleen and intestines (**Figures 1B** and **2A**), suggesting LPS-induced inflammation was not strictly localized to the injection site. This however, is expected. LPS is ubiquitous in the lumen of the gut and has been shown to induce IL-12 production *in vitro* (30–33). Increased IL-12 production by lamina propria mononuclear cells (LPMC) has also been observed in the intestines during models of inflammatory bowel disease upon LPS administration (34). Regarding IL-12 production in the spleen, LPS treatment alone does not induce IL-12 production *in vitro*. However, in combination with IFNγ, LPS does induce IL-12 production in fresh spleen cells (35). This likely explains our results *in vivo*. High background levels of tracer accumulation can be seen in organs with high blood content such as liver and lung. High background levels of tracer may limit the efficacy of this imaging approach in specific sites. Site specific studies may be required to fully understand the limitations of this technology.

### [^89^Zr]Zr-DFO-αIL12 Tracer Identifies Localized IL-12 Production in Tumors Treated With Immune-Stimulating Virus

**Figure 3:**
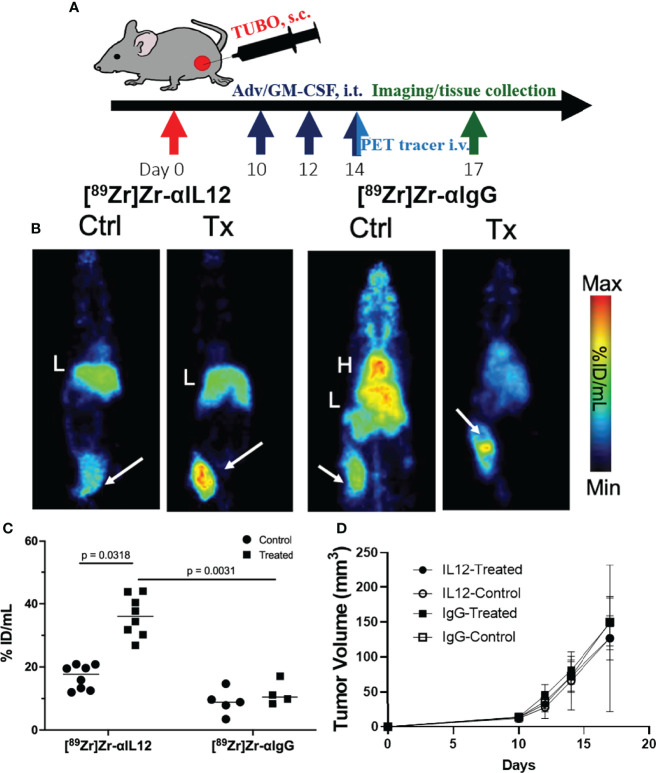
Detection of localized IL-12 production in the tumor microenvironment **(A)**. Treatment and PET imaging scheme **(B).** Representative maximum intensity projection images of Adv/GM-CSF treated vs. untreated mice administered with [^89^Zr]-αIL-12 tracer or [^89^Zr]-αIgG; tumors are indicated by white arrows **(C).** Tumor uptake of the tracers (%ID/mL) comparing control versus treated cohorts **(D).** Tumor volume progression over time until tissue harvest. H, Heart; L, Liver.

We next evaluated the utility of the [^89^Zr]Zr-DFO-αIL12 tracer as a measure of localized cancer immunotherapy response where antigen presenting cells are activated (36). BALB/c mice bearing orthotopic TUBO mammary carcinoma tumors received intratumoral delivery of Adv/GM-CSF, which we and others have previously shown promotes anti-tumor immunity (25). TUBO tumors are known to harbor high levels of macrophage infiltrates *in vivo* (37), and M1 macrophages are known to produce IL-12 in response to GM-CSF (38). Mice were inoculated with TUBO cells and once tumors reached ~50 mm^3^ (day 10 after inoculation) received three intratumoral injections of 10^8^ PFU Adv/GM-CSF, or vehicle control, once every two days (**Figure 3A**). On the last day of treatment, each subject received tracer injection of either [^89^Zr]Zr-DFO-αIL12 or [^89^Zr]Zr-DFO-αIgG isotype control. It should be noted that IL-12 and IgG control experiments were performed separately due to limitations surrounding radiotracer production and imaging suite capacity. All mice were imaged at 72 h p.i. *via* PET. Representative planar images (**Figure 3B**) and tumor uptake (**Figure 3C**) demonstrate significantly higher uptake of [^89^Zr]Zr-DFO-αIL12 in treated vs. untreated mice (47.3 ± 9.4%ID/g vs. 20.9 ± 5.4%ID/g, p = 0.0002). [^89^Zr]Zr-DFO-αIgG uptake is not statistically different between treated and untreated mice (11.6 ± 4.7%ID/g vs. 8.5 ± 1.0%ID/g, p = 0.21). Accumulation of the IL12p70 tracer is significantly higher versus the IgG control tracer in the treated groups (p = 0.0016), suggesting uptake is not induced by enhanced permeability and retention effects. A closer analysis revealed a small but significant elevation in tracer uptake in tumor-bearing female versus male hosts (**Figure S2**), implying there may be differences in magnitude of response based on biological sex. However, because of the relatively small cohort (n=4 for each), further studies are required with a larger sample size to confirm biological relevance.

### [^89^Zr]Zr-DFO-αIL12 Uptake Correlates With Activated Intratumoral Antigen Presenting Cells After Adv/GM-CSF

**Figure 4:**
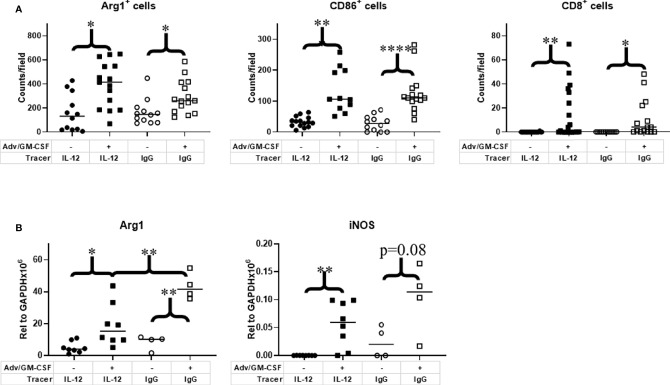
Analysis of immune cell infiltrates in the tumor microenvironment. Tumor tissue collected at 1 week post Adv/GM-CSF administration, 3 days post [^89^Zr]-αIL-12/ [^89^Zr]-αIgG control administration. **(A)** IHC enumeration of paraffin embedded slides, select tumor infiltrating cell populations, 3 highest density fields per sample. M1 Macrophage (CD86). M2 Macrophage (Arg1). CD8+ Tc (Cytotoxic T cells). **(B)** qRT-PCR analysis of M1 (iNOS) and M2 (Arg1) signature genes in flash frozen tumor tissue. *p value < 0.05, **p value < 0.01, ****p value < 0.001.

The primary aim of this study was not to evaluate treatment impact on tumor growth, as our previous study unsurprisingly showed no observable difference in volume at the imaging timepoint after Adv/GM-CSF treatment (**Figure 3D**) (25). There are, however, notable differences in the TME. Immediately after IL-12 or IgG control PET imaging, tumors were harvested and formalin-fixed tissues were analyzed by immunohistochemistry (IHC, **Figure 4A**). Three 20x high-density fields were taken of each tumor section, and Arg1 (as a marker of M2 TAMs), CD86 (as a marker of M1 TAMs), and CD8 (CD8^+^ T_c_) positivity was evaluated using AI trainable WEKA segmentation. Each marker was evaluated *via* two-way ANOVA. In each case, it was shown that Adv/GM-CSF treatment significantly increased the evaluated tumor infiltrating cell populations. No populations evaluated by IHC were significantly different between the experimental IL-12 and control IgG PET tracers (**Figures 4A** and **S3–S8**). CD86^+^ cell counts in Adv/GM-CSF treated tumors that received [^89^Zr]Zr-DFO-αIL12 outnumber CD86^+^ populations in untreated tumors by over 3-fold (Tx mean cells/field = 132 +/- 70 vs. UTx mean cells/field = 33 +/- 17, p < 0.0001) (**Figure S3**). We also find a higher abundance of Arg1^+^ infiltrates by over 2-fold (Tx mean cells/field = 395 +/- 187 vs. UTx mean cells/field = 159 +/- 153, p = 0.0003) (**Figure S4**), suggesting abundance of M2-polarized macrophages may also increase with treatment. IL-12 production is also known to support CD8 T cell proliferation (39), and we find increased CD8^+^ infiltrates in Adv/GM-CSF treated tumors (Tx mean cells/field = 13 +/- 21 vs. UTx mean cells/field = 0.05 +/- 0.22, p = 0.006) (**Figure S5**). Representative scanned IHC images and AI assisted enumeration can be found in the supplement (**Figures S3–S8**).

As an additional method to investigate macrophage polarization, we also evaluated flash frozen tumor sections for iNOS (M1) and Arg1 (M2) transcripts by qRT-PCR. Largely, we find the qRT-PCR data closely mirrors our IHC results (**Figure 4B**). iNOS expression, an alternative M1 marker, was significantly higher in Adv/GM-CSF-treated tumors that received [^89^Zr]Zr-DFO-αIL12 tracer (p = 0.0016). Although iNOS expression was marginally insignificant between Adv/GM-CSF treated and untreated tumors (p = 0.08). Arg1 expression evaluated *via* qRT-PCR yielded similar results. Mice receiving either tracer exhibited higher Arg1 expression after receiving Adv/GM-CSF administration (IL-12, p = 0.0125; IgG control, p = 0.0003).

The only evidence that tracer may be interfering with downstream IL-12 signaling is that significantly higher Arg1 expression is observed at the RNA level in tumors that received IgG control tracer as compared to the experimental IL12 tracer (p = 0.0079). While this change is observable at the RNA level, this is not reflected in phenotypic analysis displayed by our IHC results (**Figures 4A, B****)**. This observation led us to closely consider downstream elements of IL-12 signaling.

### A Single Dose of [^89^Zr]Zr-DFO-αIL12 Tracer Has No Discernable Impact on IL-12 Signaling

**Figure 5:**
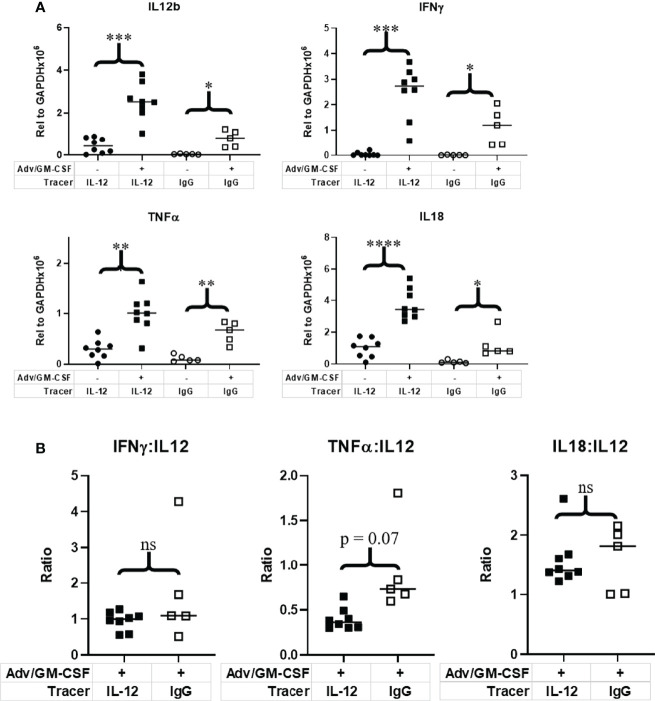
Evaluation of IL-12 and downstream immune signaling in the tumor microenvironment. Tumor tissue collected at 1 week post Adv/GM-CSF administration, 3 days post [^89^Zr]-αIL-12/ [^89^Zr]-αIgG control administration. qRT-PCR analysis of **(A)** IL-12 and downstream signaling genes evaluated in flash frozen tumor tissue. **(B)** Relative expression of IL-12 signaling genes to IL-12 expression. *p value < 0.05, **p value < 0.01, ***p value < 0.001, ****p value < 0.001, ns, not significant.

To validate expression of IL-12 and test whether tracer administration impacts downstream IL-12/IL-12R signaling, we performed qRT-PCR on flash frozen tumor tissue to measure expression of IL-12β and key proinflammatory cytokines indicative of active IL-12 signaling: IFNγ, TNFα, and IL-18 (**Figure 5A**). Mean IL-12β expression relative to GAPDH was increased post Adv/GM-CSF treatment with both the IL-12 (p = 0.0001) and IgG (p = 0.003) PET tracer cohorts. Interestingly, IL-12 expression was also higher in mice receiving [^89^Zr]Zr-DFO-αIL12 versus [^89^Zr]Zr-DFO-αIgG (p = 0.004) (**Figure 5A**). We do not believe this to be a biologically relevant observation, and attribute this primarily to unknown batch variation between cohorts. In order to better compare downstream signaling between [^89^Zr]Zr-DFO-αIL12 and [^89^Zr]Zr-DFO-αIgG control treated mice, we compare relative expression of signaling genes to IL-12 expression. Both IFNγ and IL-18 signaling were not significantly different between [^89^Zr]Zr-DFO-αIL12 and [^89^Zr]Zr-DFO-αIgG control mice. A marginal but non-statistically significant difference can be seen between [^89^Zr]Zr-DFO-αIL12 and [^89^Zr]Zr-DFO-αIgG control mice in the case of TNFα signaling (**Figure 5B**). TNFα signaling is modestly reduced in [^89^Zr]Zr-DFO-αIL12 treated mice (p = 0.07) as compared to [^89^Zr]Zr-DFO-αIgG controls. These observations suggest that [^89^Zr]Zr-DFO-αIL12 permits downstream IL-12 signaling without major interference by the tracer antibody itself.

## Discussion

Activation of APCs often precedes adaptive immune induction. Thus, IL-12 may give a snapshot of immune function preceding lymphocyte activation compared to other tracers targeting immune molecules associated with adaptive immunity, including granzyme B and IFNγ (6, 7). Additionally, production of IL-12 is generally associated with activation of CD8^+^ T_c_, the T_h1_ lineage of CD4^+^ T cells, and a more M1-like macrophage environment (15, 16, 20). Detection of IL-12 may thus serve as an indicator of the overall immune milieu.

IL-12 has long been considered a potential inroad for therapeutic intervention. Its involvement in inflammation makes it an attractive target for non-invasive imaging through PET. This [^89^Zr]Zr-DFO-αIL12 tracer represents a potentially useful tool in monitoring inflammation during immunotherapy or other chronic inflammatory conditions. To the best of our knowledge, this is the first immunoPET radiotracer reported to target IL-12 *in situ*. We have demonstrated that [^89^Zr]Zr-DFO-αIL12 successfully delineated LPS-induced inflammation, a highly immunogenic compound known to be involved in systemic bacterial infection. [^89^Zr]Zr-DFO-αIL12 tracer accumulation at the LPS injection site was significantly higher compared to the C.L. muscle as well as in unchallenged mice. In addition, we have shown the capability of this tracer to detect an active inflammatory response elicited during Adv/GM-CSF immunotherapy.​ Tumors treated in this fashion more efficiently recruit macrophage as well as effector CD8^+^ T_c_ cells (**Figure 4A**). Importantly, this effect is not abrogated by the administration of IL-12 tracer. While untested, there is the potential for tracer binding receptor-bound, as well as free IL-12. There is a small significant decrease in Arg1 mRNA levels in tumors that received IL-12 tracer as compared against the IgG control, however this difference is likely transient. There is no difference at the protein level, as determined *via* IHC. This observation led us to investigate other downstream elements of IL-12 signaling axis *via* qPCR. We find that IL-12 signaling remains intact in these tumor tissues despite the potential for Ab depletion of cytokine, and/or off target F_c_R-F_c_ interactions. This was evidenced by elevated levels of canonical IL-12 signaling genes in Adv/GM-CSF treated tumors, as detected *via* qPCR and when comparing transcripts from tumors imaged with IL-12 vs. IgG control tracers. Collectively, these findings establish that there is marginal to no significant disruption of IL-12 signaling that can alter overall immune function when [^89^Zr]Zr-DFO- αIL12 is used for detection.

While systemic administration of IL-12 as an immunotherapy has proven toxic (40, 41), other more targeted approaches are under investigation to locally induce IL-12. These include priming of CD8^+^ T cells prior to adoptive transfer (42), Ab-targeted nanoparticles containing IL-12 (43), delivery of IL-12 *via* CAR T cells (44), and genetically engineered CD8^+^ T_c_ to produce single-chain IL-12 prior to adoptive transfer (22). Approaches to neutralize IL-12 signaling have also had some success in the treatment of chronic inflammatory conditions. Ustekinumab and Briakinumab are two such examples of humanized mAb targeting subunit p40, shared by both IL-12 and IL-23 (45, 46). Depletion strategies have been shown to be effective in a variety of inflammatory autoimmune disorders, such as plaque psoriasis and psoriatic arthritis (47–50), as well as Crohn’s disease and ulcerative colitis (51–53). Clinical decisions on whether these immune modulation approaches are efficacious in patients can potentially be guided by the use of the IL-12 tracer. The ultimate goal is to move this imaging technique into human subjects now that proof of concept is achieved. An anti-human p70-specific antibody clone would be required for IL-12 specificity. Humanized mice or genetically engineered models can be utilized for pre-clinical testing. Toxicity studies would be required prior to implementation in humans, though the effects of similar antibodies are well documented.

Divorced from the IL-12 molecule itself, PET imaging against other soluble immune signaling molecules could provide real benefits for patients receiving depleting mAb therapeutics, such as ustekinumab in the case of Crohn’s disease. Patients taking these therapeutics are often caught in an indefinite monthly cycle of costly treatments. PET imaging technology could aid in determining the need for continuation of the depleting mAb therapy. Tools to non-invasively monitor localized immune function *in vivo* have the potential to improve patient care, particularly for cancer immunotherapy and chronic inflammatory conditions including autoimmunity. Recent emerging biologic immune modulating therapies are expensive, and particularly in the case of cancer immunotherapy, frequently induce high-grade immune-related adverse side effects. Imaging technologies including the [^89^Zr]Zr-DFO-αIL12 tracer presented here have the potential to guide clinical decision-making and support personalized individual treatment regimens.

## Data Availability Statement

The original contributions presented in the study are included in the article/**Supplementary Material**. Further inquiries can be directed to the corresponding authors.

## Ethics Statement

The animal study was reviewed and approved by Wayne State University Animal Care and Use Committee.

## Author Contributions

NV and HG conceived of the theoretical basis for the implementation of radiotracer to image IL-12. *In vivo* work was performed by HG, JG, JK, and JH. Radiotracer labeling and imaging was performed by JK, JS, and MW. Imaging analysis was performed by NV. Ex vivo work (IHC and enumeration, qPCR, data analysis) was performed by JG. Manuscript was written by JG with editing by HG and NV. All authors contributed to the article and approved the submitted version.

## Funding

The authors acknowledge the Microscopy, Imaging and Cytometry Resources Core, which is supported in part by the National Institutes of Health (NIH) grant P30 CA022453 to the Karmanos Cancer Institute at Wayne State University. Research was funded in part by the National Cancer Institute under the NIH. Award numbers include R37 CA220482 (NV and HG), and the Ruth L. Kirschstein National Research Service Award T32-CA009531 (JG).

## Conflict of Interest

NV and HG are inventors on U.S. Provisional Patent Application Serial No. 62/888,747 “*In Vivo* Immunoimaging of Interleukin-12”.​

The remaining authors declare that the research was conducted in the absence of any commercial or financial relationships that could be construed as a potential conflict of interest.

## Publisher’s Note

All claims expressed in this article are solely those of the authors and do not necessarily represent those of their affiliated organizations, or those of the publisher, the editors and the reviewers. Any product that may be evaluated in this article, or claim that may be made by its manufacturer, is not guaranteed or endorsed by the publisher.
