# Supplementary material for: Evaluation of an ImmunoPET Tracer for IL-12 in a Preclinical Model of Inflammatory Immune Responses
Source: Front Immunol. 2022 May 11;13:870110. doi: 10.3389/fimmu.2022.870110 (PMC9130849; doi:10.3389/fimmu.2022.870110)
Supplement: Supplementary file 1 [file DataSheet_1.pdf]

Supplementary Material

1 Supplementary Figures

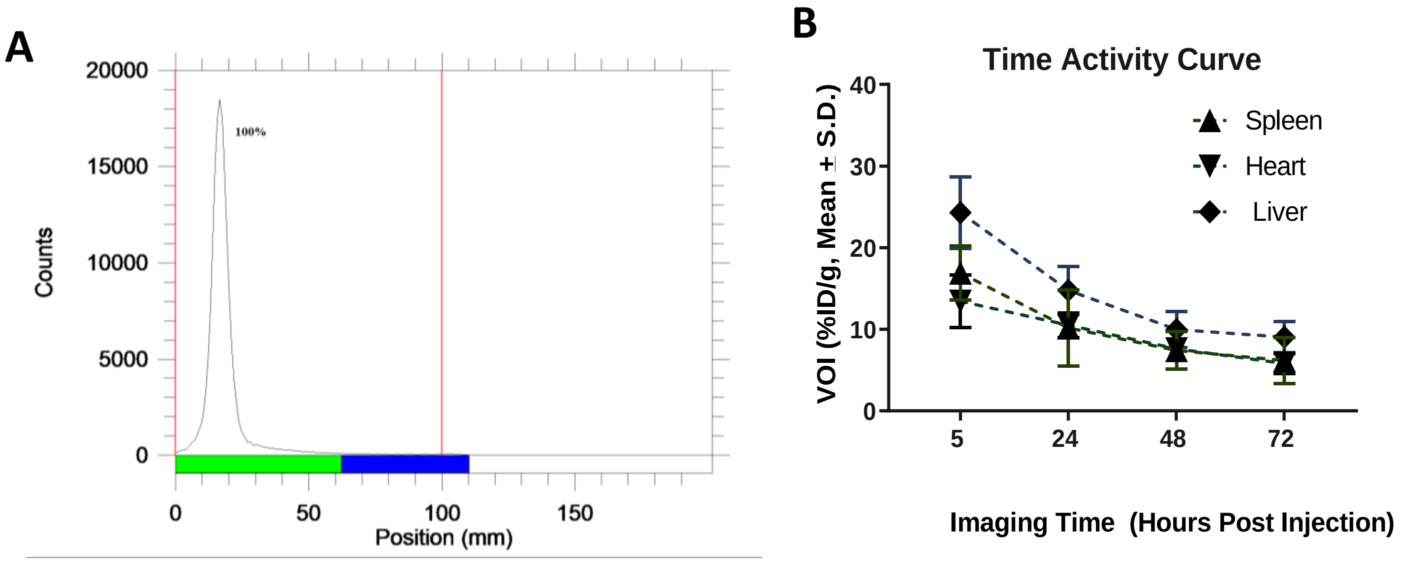

S1. A. Representative iTLC chromatogram showing purity of [^{89}Zr]Zr-DFO-αIL12 **B.** Uptake of [^{89}Zr]Zr-DFO-αIL12 in Spleen, Heart, and Liver tissue over time.

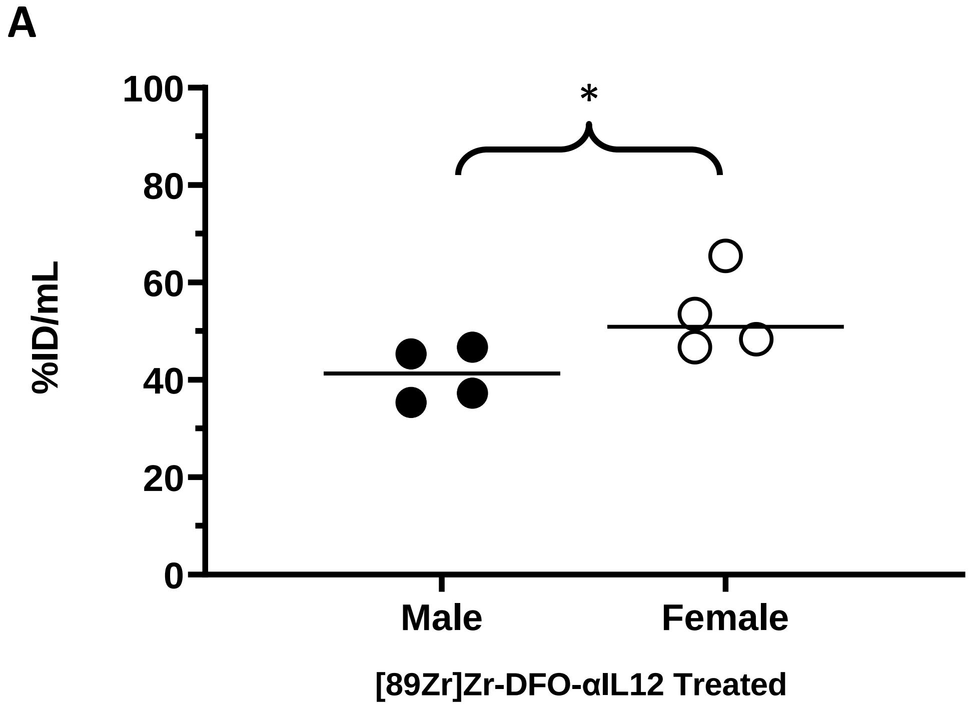

S2. Differences in biological sex. A. [^{89}Zr]Zr-DFO-αIL12 Tracer uptake in %ID/mL by biological sex.

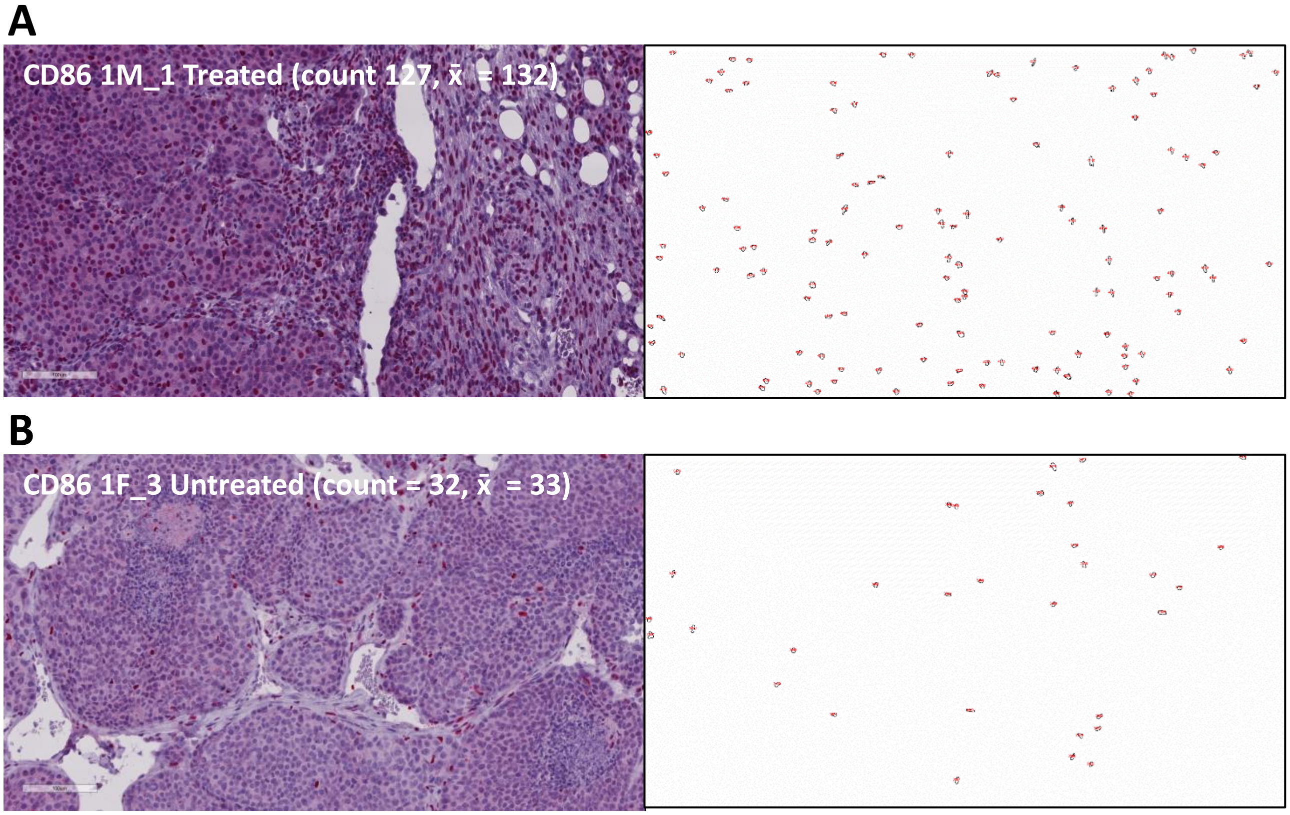

S3. Immunohistochemistry of paraffin embedded, oncolytic Adv/GM-CSF Treated/Untreated tumors (CD86), 3 days post [^{89}Zr]Zr-DFO-αIL12 administration. A. Representative treated tissue section, count as determined by AI trainable Weka segmentation, and mean counts per field. **B.** Representative untreated tissue section, count as determined by AI trainable Weka segmentation, and mean counts per field.

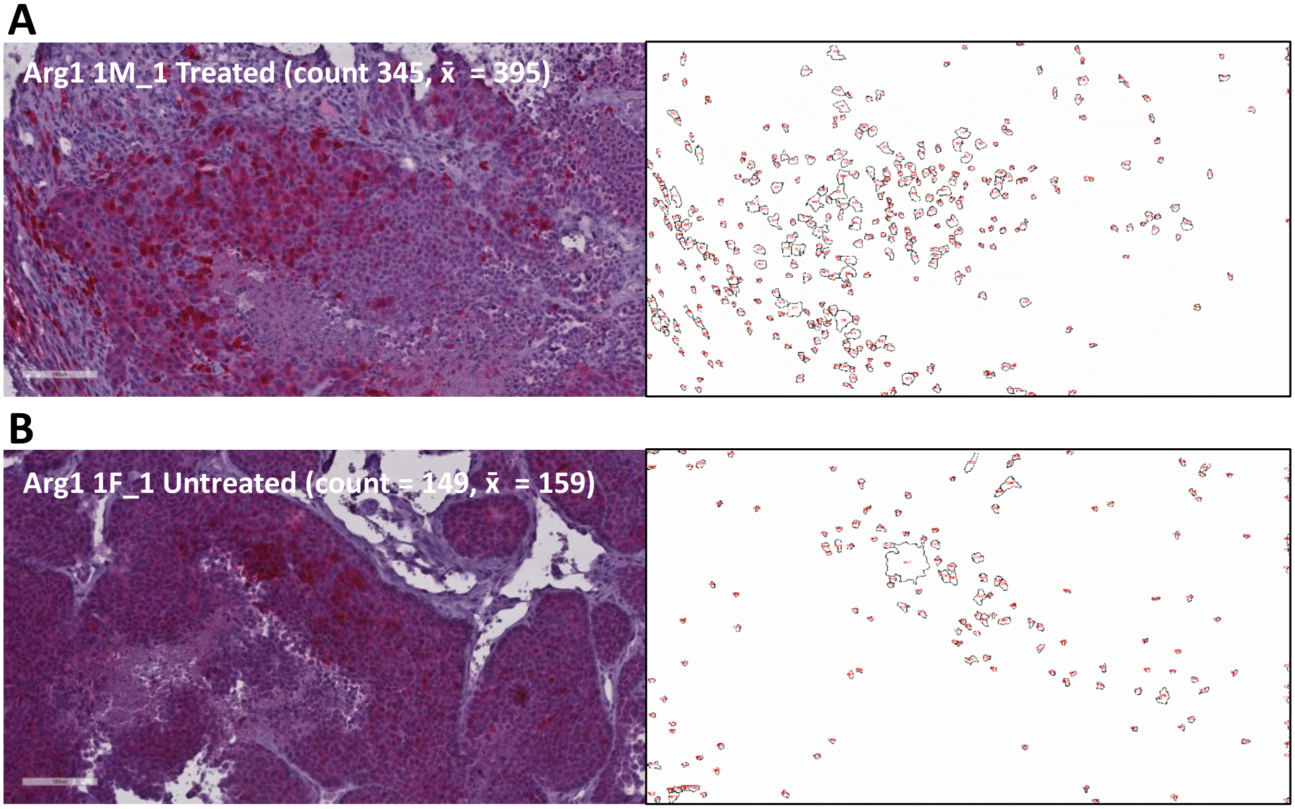

S4. Immunohistochemistry of paraffin embedded, oncolytic Adv/GM-CSF Treated/Untreated tumors (Arg1), 3 days post [^{89}Zr]Zr-DFO-αIL-12 administration. A. Representative treated tissue section, count as determined by AI trainable Weka segmentation, and mean counts per field. **B.** Representative untreated tissue section, count as determined by AI trainable Weka segmentation, and mean counts per field.

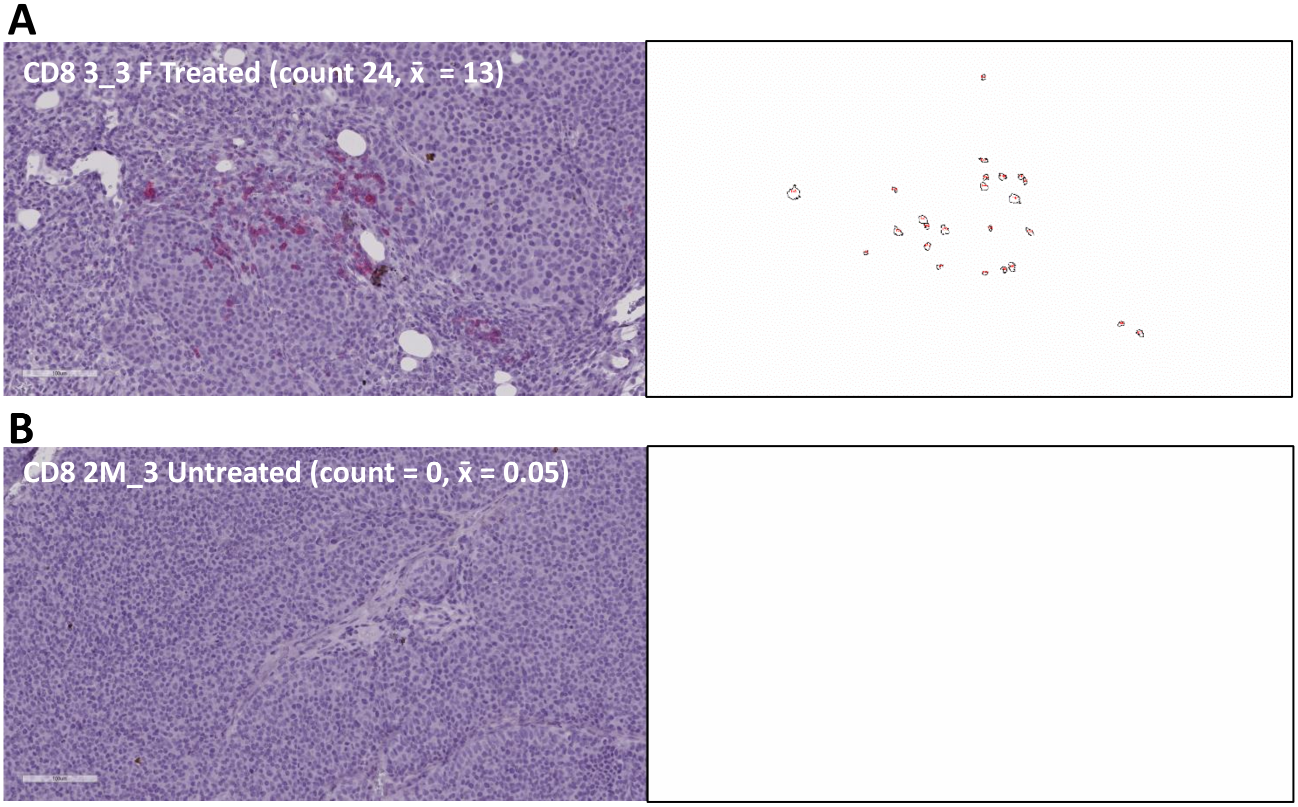

S5. Immunohistochemistry of paraffin embedded, oncolytic Adv/GM-CSF Treated/Untreated tumors (CD8), 3 days post [^{89}Zr]Zr-DFO-αIL12 administration. A. Representative treated tissue section, count as determined by AI trainable Weka segmentation, and mean counts per field. **B.** Representative untreated tissue section, count as determined by AI trainable Weka segmentation, and mean counts per field.

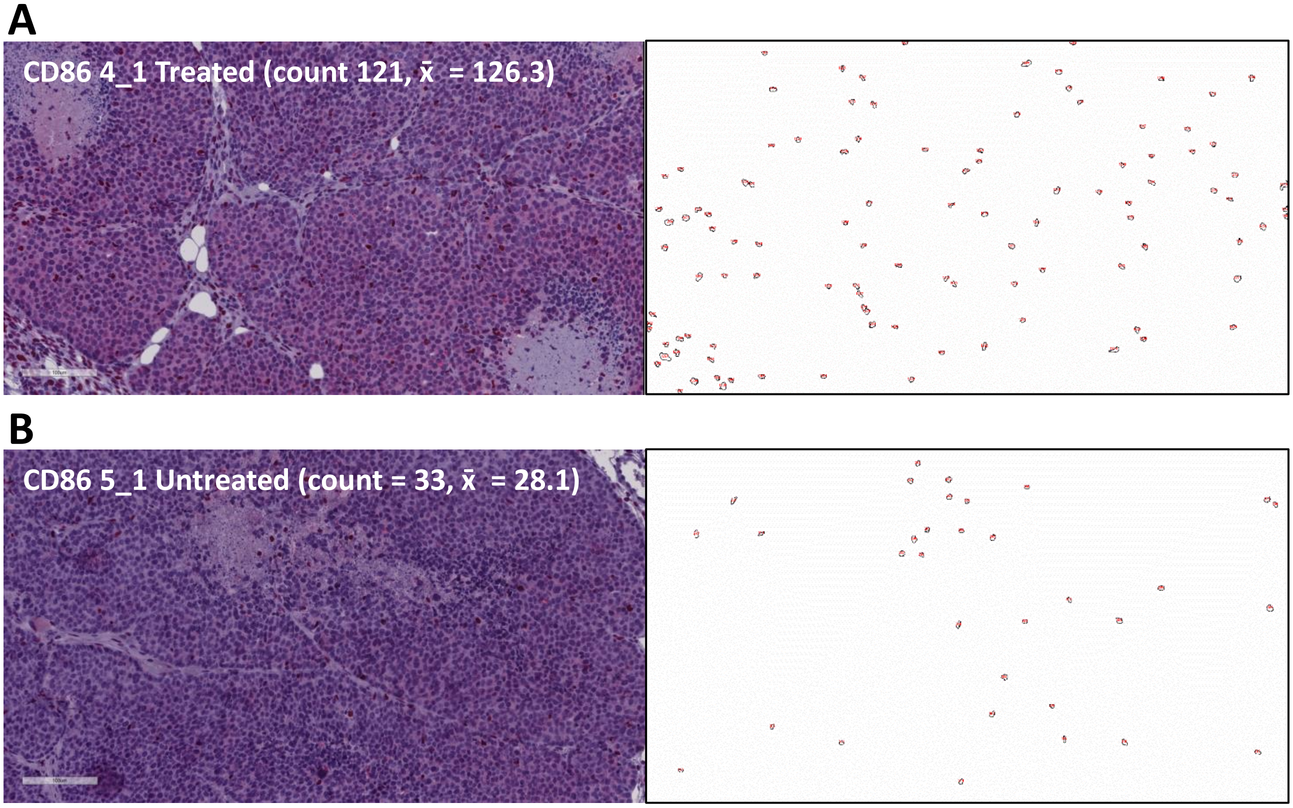

S6. Immunohistochemistry of paraffin embedded, oncolytic Adv/GM-CSF Treated/Untreated tumors (CD86), 3 days post [^{89}Zr]-αIL-IgG administration. A. Representative treated tissue section, count as determined by AI trainable Weka segmentation, and mean counts per field. **B.** Representative untreated tissue section, count as determined by AI trainable Weka segmentation, and mean counts per field.

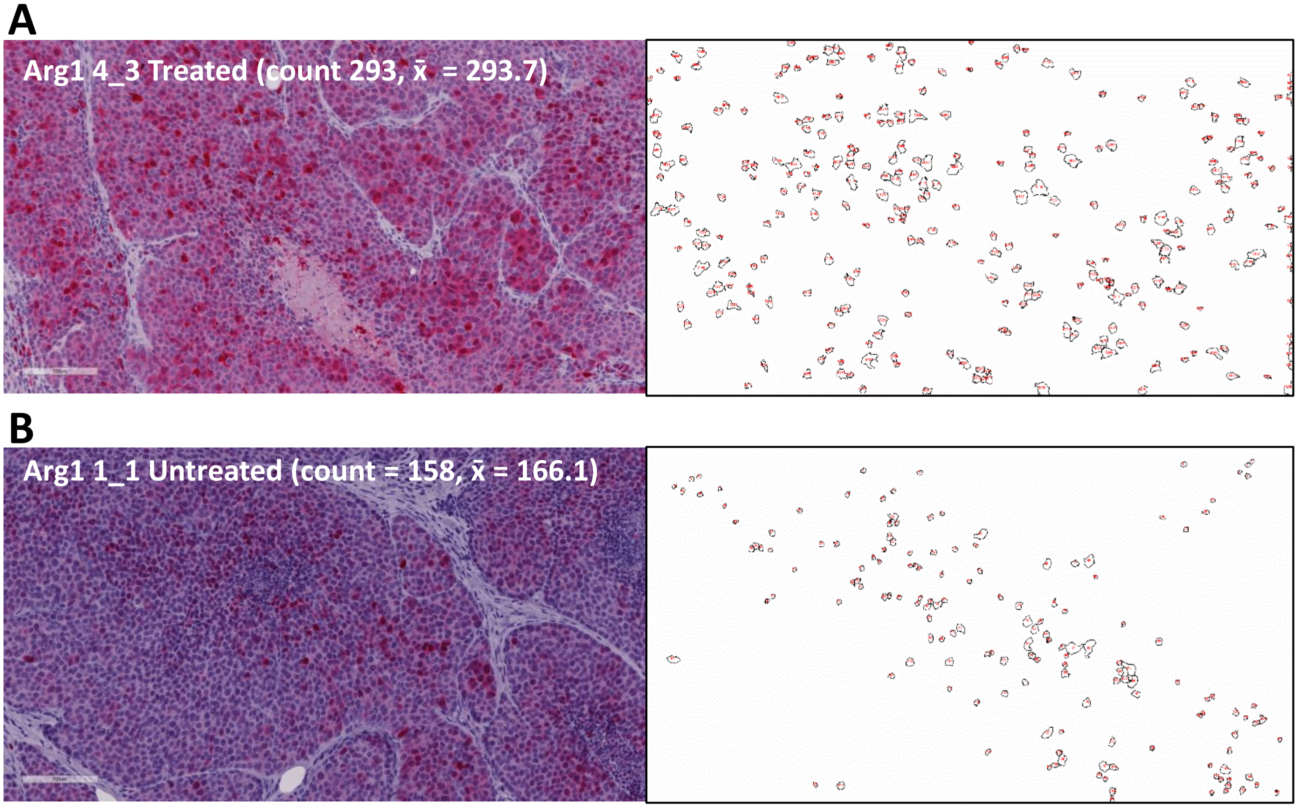

S7. Immunohistochemistry of paraffin embedded, oncolytic Adv/GM-CSF Treated/Untreated tumors (Arg1), 3 days post [^{89}Zr]Zr-DFO-αIgG administration. A. Representative treated tissue section, count as determined by AI trainable Weka segmentation, and mean counts per field. **B.** Representative untreated tissue section, count as determined by AI trainable Weka segmentation, and mean counts per field.

A

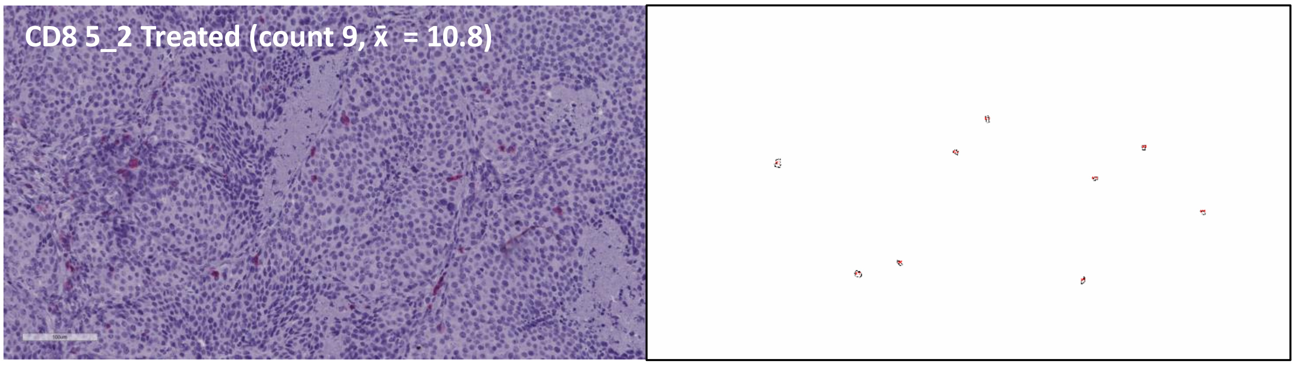

B

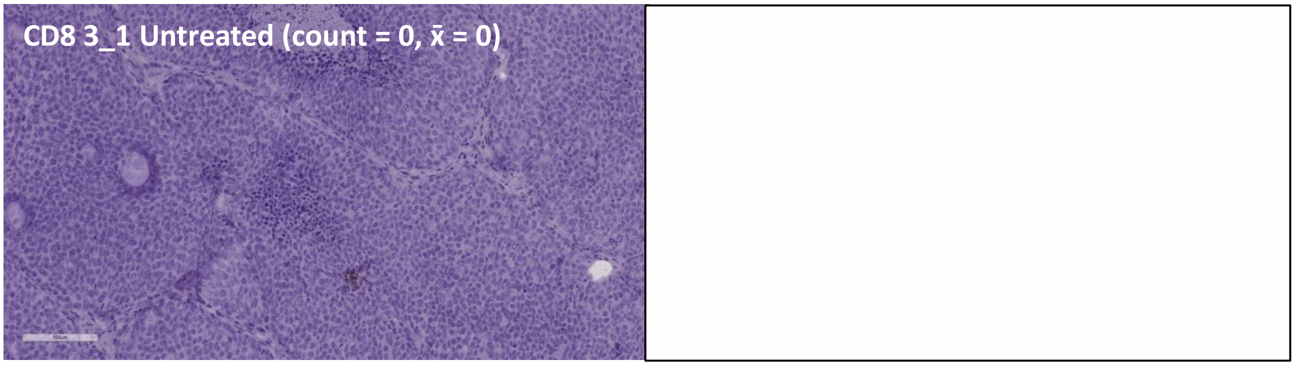

S8. Immunohistochemistry of paraffin embedded, oncolytic Adv/GM-CSF Treated/Untreated tumors (CD8), 3 days post [^{89}Zr]Zr-DFO-αIgG administration. A. Representative treated tissue section, count as determined by AI trainable Weka segmentation, and mean counts per field. **B.** Representative untreated tissue section, count as determined by AI trainable Weka segmentation, and mean counts per field.

**Table S1.Stability of [^{89}Zr]Zr-DFO-αIL12 in saline at 37 °C.**	
Time (h)	%Intact (n = 1)
0	100 %
24	99 %
48	98 %
72	98 %
96	98 %
